# Metabolic disorders in young people around the world

**DOI:** 10.1007/s00125-025-06450-2

**Published:** 2025-06-17

**Authors:** Sirisha Kusuma Boddu, Cosimo Giannini, M. Loredana Marcovecchio

**Affiliations:** 1 Department of Pediatric Endocrinology and Diabetes, Rainbow Children’s Hospital, Hyderabad, Telangana India; 2 Department of Paediatrics, University of Chieti, Chieti, Italy; 3 Department of Paediatrics, University of Cambridge, Cambridge, UK; 4 Department of Paediatric Endocrinology and Diabetes, Cambridge University Hospitals NHS Foundation Trust, Cambridge, UK

**Keywords:** Cardiometabolic, Equity, diversity and inclusion, Obesity, Review, The metabolic syndrome, Type 1 diabetes, Type 2 diabetes, Youth

## Abstract

Youth-onset metabolic diseases, including obesity and type 1 and type 2 diabetes, and their associated cardiometabolic complications represent a major global health challenge. The incidence and prevalence of these conditions vary across regions, with rising trends and a heavier burden observed in middle- and low-income countries. Diet, physical activity and lifestyle choices are key factors in the development and progression of metabolic diseases during childhood and adolescence, along with additional risk factors such as genetic predisposition, ancestry, ethnicity, lifetime events (i.e. puberty) and other environmental factors. Disparities in access to healthcare, diagnostic and management capabilities and treatment options across the world affect outcomes, contributing to high morbidity and mortality rates, particularly in low-resource settings. Compared with onset during adulthood, an early diagnosis of metabolic diseases is associated with a higher risk and severity of complications, including adverse vascular outcomes and premature mortality. Although clinical signs of cardiovascular complications typically appear in adulthood, they are the result of a long, subclinical disease process that can begin in childhood and adolescence. This underscores the need for early prevention strategies and effective treatments to reduce the short- and long-term health impacts of these conditions. Addressing the rising prevalence of metabolic diseases, especially among vulnerable populations, requires comprehensive, culturally tailored actions that consider the available resources in diverse settings.

**Graphical Abstract:**

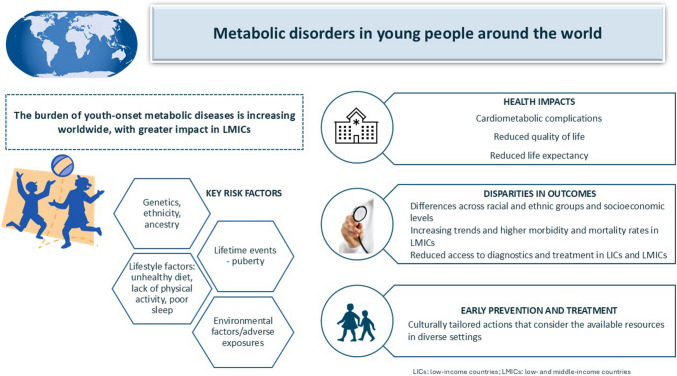

**Supplementary Information:**

The online version contains a slideset of figures for download available at 10.1007/s00125-025-06450-2.

## Introduction

Metabolic diseases such as obesity and diabetes, along with the associated cardiometabolic complications, represent a significant global health burden, particularly among young people [1–3]. Early onset of these conditions contributes to reduced quality of life, increased morbidity and premature death, and has high healthcare costs and intergenerational effects. The prevalence and incidence of metabolic diseases have increased over the past two to three decades, largely driven by rapid economic development and environmental and societal changes [1–3]. There are notable regional and socioeconomic disparities in incidence and prevalence, with rising trends and a higher burden observed in low- and middle-income countries (LMICs). Furthermore, variation in the epidemiology of metabolic diseases is linked to ancestry and ethnicity [4, 5].

**Fig. 1:**
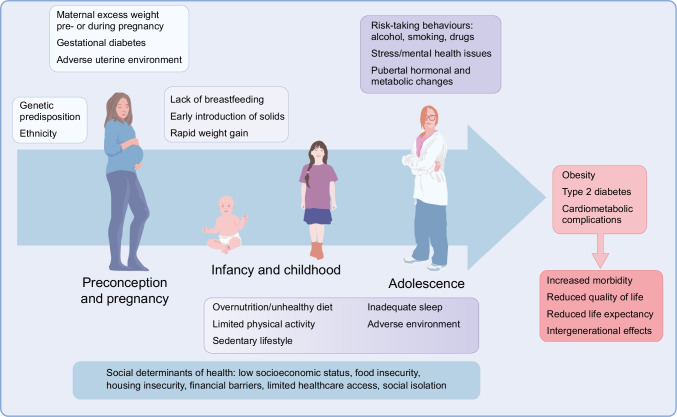
Life course of metabolic diseases in young people. The figure summarises the main risk factors acting during prenatal life, infancy, childhood and adolescence that contribute to the development of metabolic diseases, such as obesity and diabetes, as well as associated cardiometabolic complications. This figure is available as part of a downloadable slideset

Childhood and adolescence are crucial developmental stages marked by significant biological and psychosocial changes [6, 7]. These changes can influence the risk of developing metabolic diseases and related complications, as well as their management (Fig. 1). Puberty, in particular, is accompanied by several hormonal and metabolic changes, including a physiological decrease in insulin sensitivity and alterations in cardiometabolic risk factors [8]. Evidence suggests that, in children with obesity, puberty can influence the transition from a metabolically healthy status to an unhealthy status [9]. It is also a well-known risk factor for type 2 diabetes and may act as a trigger for type 1 diabetes, whose peak incidence occurs between the ages of 10 and 14 years [10]. Notably, early signs of cardiometabolic complications related to diabetes often emerge during puberty [11]. Adolescence is also a phase of life characterised by a growing desire for independence and autonomy, often accompanied by risk-taking behaviours (drugs, alcohol and smoking). These behaviours can contribute to an increased risk of complications and suboptimal adherence to lifestyle and medical interventions [12]. However, childhood and adolescence also present unique opportunities to improve metabolic health [6, 7]. These developmental stages of life provide a critical window for implementing preventive interventions targeting unhealthy behaviours such as poor diet, excessive energy intake, insufficient physical activity, inadequate sleep, and smoking and alcohol use, while also promoting mental health. These early interventions have the potential to yield long-term health benefits [7, 13].

This review provides an overview of common metabolic diseases, with a focus on youth-onset obesity and type 1 and type 2 diabetes and related cardiometabolic complications. It discusses geographical and socioeconomic variations in the incidence, management and burden of these conditions, alongside the role of ancestry and ethnicity in shaping their epidemiology and outcomes. The review also emphasises the importance of early prevention strategies and effective treatment approaches to mitigate the short- and long-term health impacts of these conditions. With regard to the terminology used in this review to distinguish populations (‘ancestry’, ‘ethnicity’ and ‘race’), when referring to previously published work we use the terms used by the authors.

## Worldwide trends in childhood obesity and associated cardiometabolic risk factors

The prevalence of overweight and obesity in children and adolescents has increased significantly in recent decades, making it a major global public health concern [14]. According to the WHO, in 2022, 37 million children under the age of 5 years and over 390 million children and adolescents aged 5–19 years were overweight or living with obesity [14]. A recent systematic review and meta-analysis including data from over 45 million children and young people from 154 different countries and regions reported that approximately one in five (22.2%) children were overweight [15]. The overall prevalence of obesity was 8.5%, but there was substantial variation across geographical regions, race and ethnicity and country income, with the highest prevalences in Hispanic and Black non-Hispanic children compared with non-Hispanic White and Asian peers (Table 1).

**Table 1:** Prevalence of obesity in children and adolescents between 2000 and 2023 based on geographical region, race and ethnicity and income

Characteristic	Prevalence (95% CI) (%)
Geographical region	
Polynesia	19.45 (16.06, 23.07)
Caribbean	19.22 (15.1, 23.7)
Northern America	17.17 (16.59, 17.75)
Central America	15.85 (14.23, 17.55)
Western Asia	9.94 (9.03, 10.88)
South America	9.38 (8.24, 10.59)
Northern Africa	9.22 (7.32, 11.3)
South-Eastern Asia	8.71 (8.26, 9.17)
Southern Europe	8.42 (7.84, 9.01)
Eastern Asia	7.78 (7.24, 8.32)
Australia and New Zealand	6.99 (5.74, 8.36)
Micronesia	5.80 (3.95, 7.98)
Southern Asia	5.79 (5.17, 6.45)
Southern Africa	5.76 (4.24, 7.50)
Eastern Europe	4.58 (3.75, 5.50)
Northern Europe	4.55 (3.57, 5.63)
Central Asia	4.28 (2.46, 6.58)
Eastern Africa	4.12 (3.30, 5.04)
Western Africa	3.95 (3.13, 4.87)
Western Europe	3.79 (3.38, 4.22)
Melanesia	3.79 (1.84, 6.40)
Middle Africa	2.36 (1.83, 2.96)
Race and ethnicity	
Hispanic	23.55 (20.66, 26.56)
Black	16.64 (14.06, 19.39)
White	12.28 (11.19, 13.42)
Asian	9.97 (8.73, 11.29)
Country or region income	
High income	9.29 (8.95, 9.64)
Upper-middle income	8.50 (8.02, 8.99)
Lower-middle income	6.35 (6.09, 6.62)
Low income	3.60 (2.54, 4.83)

Data reproduced from Zhang et al [15] under the terms of the CC-BY license

The development of overweight/obesity is related to several behavioural, environmental and sociocultural influences, which represent potential targets for interventions [16–18]. While the heritability of obesity is between 40% and 70% [16, 17], early exposure to an obesogenic environment plays a key role in its development [18]. A strong link exists between maternal overweight/obesity, excessive weight gain during pregnancy, gestational diabetes, an adverse uterine environment and the risk of childhood obesity and related complications [19, 20] (Fig. 1). Postnatally, lack of breastfeeding and early introduction of complementary foods and beverages, particularly in formula-fed babies, have been associated with an increased risk of excess weight gain [18]. This risk is exacerbated by the widespread availability of ultra-processed and energy-dense foods, and physical inactivity. Of note, the recent increase in childhood obesity has been particularly pronounced among poorer populations and in rural areas in LMICs, where limited access to affordable healthy food is a major contributing factor [21]. Lack of exercise is also common in LMICs, particularly among girls and women living in urban informal settlements, because of restricted space and fewer opportunities for physical activity [22]. Moreover, children from families with lower parental education or income levels are more likely to be overweight or living with obesity [23].

The rising rates of youth-onset overweight and obesity are concerning, given their strong association with both short- and long-term complications affecting many organs and tissues. In particular, youth-onset obesity is linked to multiple cardiometabolic risk factors, including insulin resistance, type 2 diabetes, dyslipidaemia, hypertension and metabolic dysfunction-associated steatotic liver disease, from an early age [24]. Early-onset obesity is also associated with subclinical signs of cardiovascular damage, including atherosclerosis and left ventricular hypertrophy, and cardiovascular events as early as 40 years of age [25].

Cardiometabolic risk factors associated with obesity often cluster together and the concept of the metabolic syndrome is often used to define the constellation of visceral obesity, hypertension, dyslipidaemia and abnormal glucose tolerance [26].

According to a recent systematic review, the prevalence of the metabolic syndrome in youth with obesity was 29.4%, varying from 2.1% to 74.4% across studies [27]. The metabolic syndrome is an emerging issue in LMICs, where its prevalence is estimated to be between 24.1% and 56.3% among individuals with overweight and obesity compared with 3.98–8.91% in the general population, although comprehensive data from certain regions, particularly sub-Saharan Africa, are lacking [27, 28]. Racial and ethnic differences in the prevalence of individual cardiometabolic risk factors and the metabolic syndrome exist, even after accounting for environmental factors [4]. For instance, non-Hispanic Black adolescents show lower prevalences of the metabolic syndrome and its individual components than their Mexican American peers [4].

There is still a lack of consensus around the definition of the metabolic syndrome for the paediatric population and different criteria have been proposed over time, leading to variations in prevalence and incidence across different studies [26, 29]. The developmental changes occurring during childhood and adolescence also impact the long-term reliability of a diagnosis of the metabolic syndrome, with a significant proportion of individuals diagnosed in childhood no longer meeting the criteria during follow-up and when transitioning to adulthood. Considering individual cardiometabolic risk factors as continuous rather than dichotomous variables is more useful for screening, prevention and management strategies during childhood and adolescence [30].

## Youth-onset type 2 diabetes across the globe

**Fig. 2:**
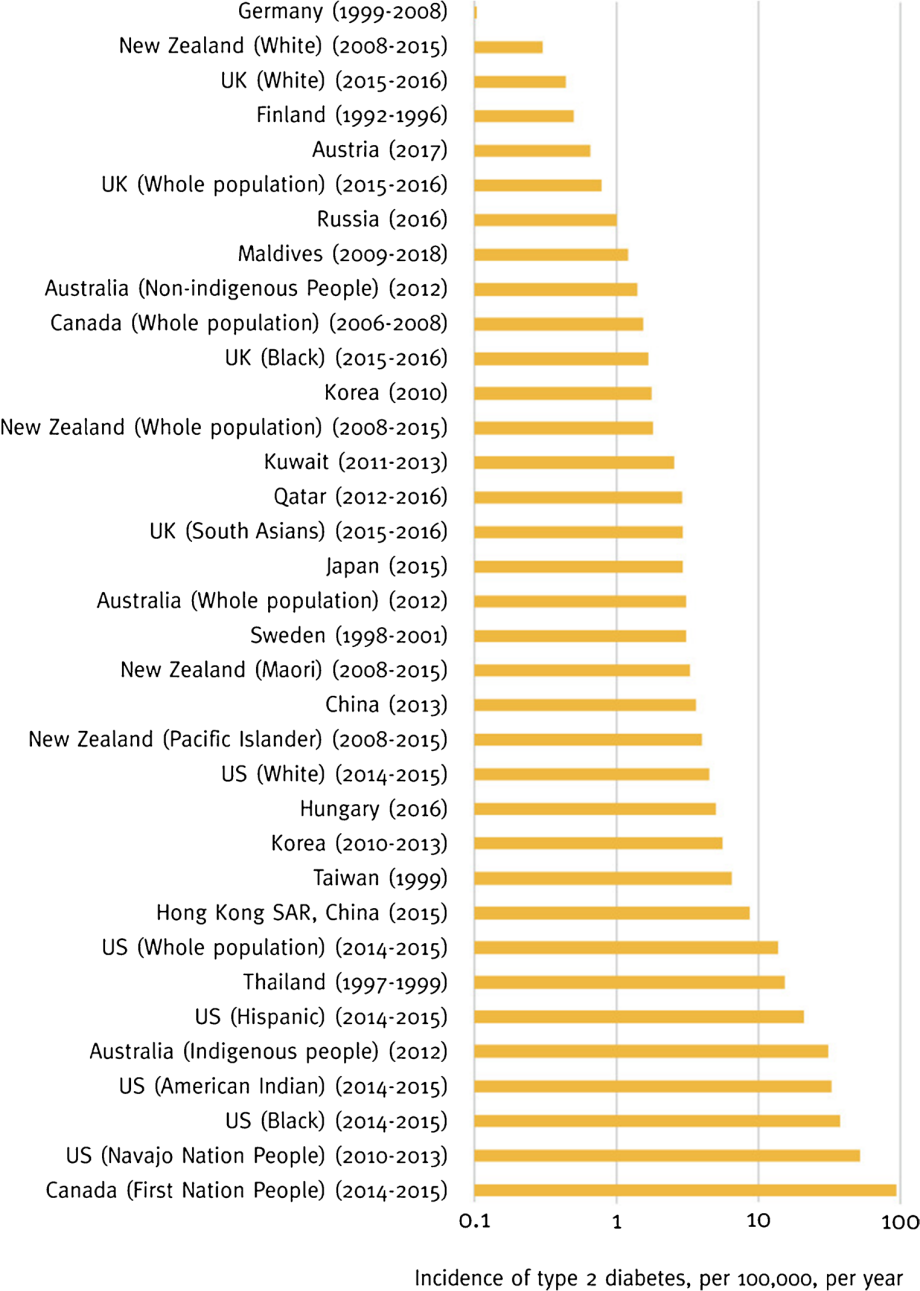
Most recent data available on the incidence of type 2 diabetes in youth ranked by region and ethnicity. Reproduced without modifications from the IDF diabetes atlas, 10th edition [39], under the terms of the CC BY-NC-ND 4.0 licence (https://creativecommons.org/licenses/by-nc-nd/4.0/). This figure is available as part of a downloadable slideset

The incidence and prevalence of type 2 diabetes in youth have increased over recent decades in many countries, although with variable rates based on region, ethnic group and socioeconomic status [31–34] (Fig. 2). The SEARCH for Diabetes in Youth study in the USA has reported a doubling in the incidence of type 2 diabetes between 2002 and 2018 [31, 32]. Increasing trends have also been observed in other parts of the world. Data from the Global Burden of Disease Study (GBD) 2019 [33] showed that, from 1990 to 2019, there were significant increases in the age-standardised incidence rate (per 100,000 population), from 117.22 to 183.36, and the age-standardised disability-adjusted life-years (DALYs) rate, from 106.34 to 149.61, for type 2 diabetes in adolescents and young adults (aged 15–39 years). Countries with a low-middle and middle sociodemographic index (SDI) had the highest age-standardised incidence rates and age-standardised DALY rates in 2019, whereas countries with a low SDI had the lowest age-standardised incidence rates but the highest age-standardised mortality rates [33]. More recent data from the multinational SWEET registry, covering the period from 2012 to 2021, confirmed a worldwide increase in type 2 diabetes in young people diagnosed before the age of 21 years [34]. The proportion of new cases rose from 3.2% in 2012/2013 to 6.0% in 2020/2021. Significant variability was observed across regions, with the lowest rates in Europe and the highest in North America. From 2012 to 2021, a notable increase was observed in Europe, Australia/New Zealand and North America, while changes in Asia/the Middle East and Africa did not reach statistical significance.

Youth-onset type 2 diabetes results from genetic, environmental and lifestyle factors that differ among individuals and populations [35] (Fig. 1). The genetic background is stronger than in type 1 diabetes, supported by the observation that 74–100% of youth presenting with type 2 diabetes have a first- or second-degree relative with the same condition [35]. While obesity is a major risk factor for type 2 diabetes, not all young people with this condition are living with obesity [35]. Of note, the prevalence of obesity is lower in Asian youth with type 2 diabetes than in other ethnicities [36], suggesting that genetic predisposition and other environmental exposures also play a role.

Early life determinants affecting the intrauterine environment, such as maternal obesity and gestational diabetes, as well as small size at birth, have been associated with an increased risk of developing type 2 diabetes [35] (Fig. 1). Youth-onset type 2 diabetes also shows significant disparities across racial and ethnic groups [37, 38], being particularly prevalent among Indigenous Australian, American Indian and Canada’s First Nation peoples [35, 39] (Fig. 2). In the Treatment Options for type 2 Diabetes in Adolescents and Youth (TODAY) study, 30% of youth were African American, 40% were Hispanic/Latino and 6% were American Indian [40]. In addition, type 2 diabetes disproportionately affects marginalised and socioeconomically vulnerable youth, with 41–59% of participants across a range of cohort studies on type 2 diabetes in youth, including TODAY and SEARCH for Diabetes in Youth, living in poverty or socially disadvantages households [41].

## Youth-onset type 1 diabetes across the globe

**Fig. 3:**
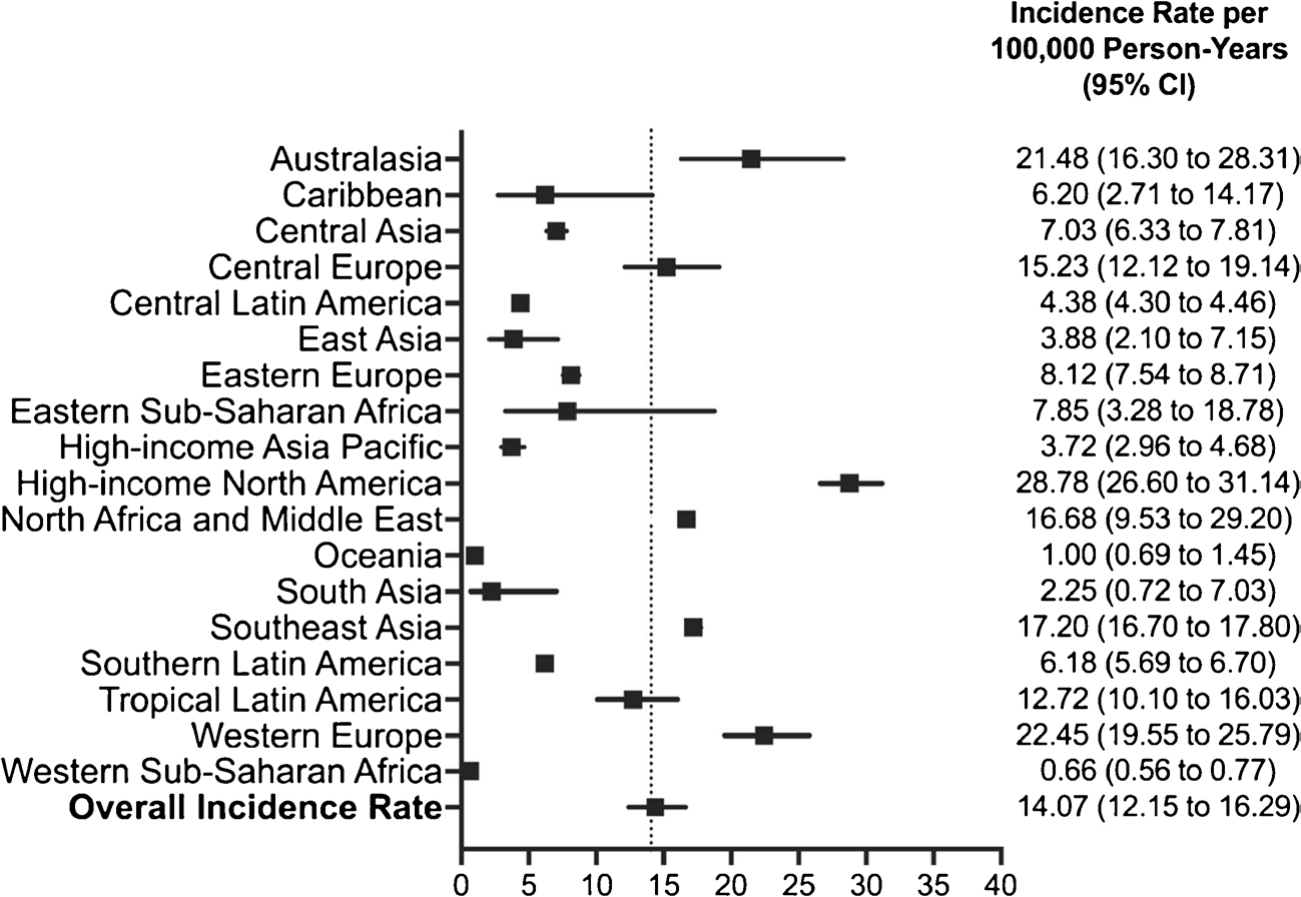
Incidence of type 1 diabetes in youth between 2000 and 2022 ranked by region. Reproduced without modifications from Hormazábal‐Aguayo et al [44] under the terms of the CC BY-NC-ND 4.0 licence (https://creativecommons.org/licenses/by-nc-nd/4.0/). This figure is available as part of a downloadable slideset

Type 1 diabetes, a chronic disorder in which autoimmune destruction of pancreatic beta cells leads to absolute life-long insulin deficiency, is the most common form of diabetes in children and adolescents [42]. According to the most recent epidemiological data from the IDF, in 2024, 9.15 million individuals worldwide were living with type 1 diabetes; of these, 1.81 million (19.8%) were aged under 20 years, with an estimated 219,000 new diagnoses [43]. A systematic review including data from 55 countries found an overall incidence rate of 14.07 per 100,000 person-years between 2000 and 2022 for those aged under 20 years, with substantial variability between countries and geographical regions. Finland and high-income North America had the highest incidence rates, at 56.81 and 28.78 per 100,000 person-years, respectively, whereas Western sub-Saharan Africa had the lowest rate, at 0.66 per 100,000 person-years [44] (Fig. 3). Alarmingly, the global burden of type 1 diabetes in youth is expected to rise substantially in the coming decades, with an expected increase in prevalence of 65% by 2060, particularly in LMICs [45, 46].

The reported epidemiological variation across countries and regions is multifactorial, with interactions between genetic predisposition and environmental factors [47]. The role of genetics in the autoimmune process leading to type 1 diabetes is supported by the concordance observed in monozygotic twins, familial clustering of this condition and findings from studies on migrant populations [47]. A comprehensive multi-ancestry genome-wide association study identified 78 chromosomal regions influencing type 1 diabetes risk, underscoring the complex genetic architecture of this condition across diverse populations [5]. Although several loci have been linked to type 1 diabetes, 30–60% of familial clustering is attributable to the HLA region [47].

Genetic factors play a role in explaining some of the differences in the prevalence of type 1 diabetes among various ethnic groups. Studies suggest that certain genetic risk factors (i.e. *HLA-DR3* and *HLA-DR4* alleles) are more prevalent in White populations, potentially contributing to higher incidence rates in these groups [48]. In contrast, the *HLA-DR7* allele may increase risk among African American populations, while *HLA-DR9* is associated with increased susceptibility in Japanese individuals [49]. In Asian populations, the susceptible *DR* alleles often occur together with neutral or even protective *DQ* alleles, probably contributing to the lower incidence rates in these regions [50].

The rapid increases in type 1 diabetes incidence and prevalence during recent decades cannot be explained by genetic alterations, which typically take much longer to manifest [47]. In addition, the increased prevalence in lower risk genotypes, especially in younger age groups, strongly favours an impact of environmental determinants [51]. Regional variations in type 1 diabetes incidence between countries, and even within some small countries with genetically homogeneous populations, also support the role of environmental factors [52]. Given the consistently high incidence of type 1 diabetes in high-income countries (HICs), socioeconomic status and nutrition and lifestyle factors might be implicated in changes in its epidemiology [53]. The rapid rise in obesity over recent decades may plausibly have an impact on the incidence of type 1 diabetes [54]. The ‘accelerator hypothesis’ implicates insulin resistance from excess weight gain and physical inactivity in hastening beta cell apoptosis in susceptible individuals [55].

In addition, lower socioeconomic status is closely linked to the achievement of less favourable glycaemic targets and reduced access to advanced diabetes care tools, including continuous glucose monitoring and hybrid closed-loop systems [56, 57]. There is also a substantially higher burden of type 1 diabetes in racial and ethnic minority young people [58]. Non-Hispanic Black and Hispanic individuals experience higher HbA_1c_ levels and lower use of diabetes technologies than their non-Hispanic White counterparts [58].

## The burden of early-onset diabetes: risks of complications and early mortality

### Risk of complications in youth with diabetes

Children and adolescents with type 1 and type 2 diabetes experience higher morbidity and mortality rates compared with the general population due to the acute and chronic complications associated with these conditions [59–61]. The long-term health burden of youth-onset diabetes is primarily driven by the associated cardiovascular complications [62], with individuals diagnosed in childhood facing a greater risk of these complications than those diagnosed in adulthood [11, 63]. This disparity is attributed to the longer diabetes duration and consequent prolonged exposure to the diabetes milieu. In addition, youth with diabetes exhibit a more aggressive disease process than adults, as reflected by more severe insulin resistance in youth with type 2 diabetes, and more rapid deterioration of pancreatic beta cell function in youth with both type 1 and type 2 diabetes [63, 64]. Furthermore, the risk of complications in this young population is affected by lifetime events such as puberty and the related hormonal changes, a decline in insulin sensitivity, psychological issues and risk-taking behaviours [65].

Although overt clinical manifestations of vascular complications, such as cardiovascular events, end-stage renal disease, proliferative retinopathy and neuropathy, typically occur in adulthood, their incidence represents the result of a long subclinical disease process starting earlier in life [66, 67]. Subclinical manifestations of vascular complications, which are often found in youth with diabetes, include increases in urinary albumin excretion (moderately increased albuminuria), background retinopathy, changes in vascular function and structure (increased carotid intima–media thickness, arterial stiffness, endothelial dysfunction) and cardiac autonomic dysfunction [11, 66, 67]. Data from the SEARCH study highlight that up to one in three youth with type 1 diabetes and nearly three in four with type 2 diabetes show evidence of early manifestations of at least one vascular complication after a mean diabetes duration of 8 years [68]. Youth with type 2 diabetes show an earlier onset of complications than those with type 1 diabetes, as well as a higher prevalence of diabetic kidney disease (20% vs 6%), retinopathy (9% vs 5.6%) and peripheral neuropathy (18% vs 8.4%), even after accounting for differences in HbA_1c_, BMI and blood pressure [68]. Cardiometabolic risk factors such as obesity, hypertension, dyslipidaemia and insulin resistance are common among adolescents with diabetes and contribute to the risk of complications [63, 68, 69]. In the SEARCH study, 15% of youth with type 1 diabetes had high triglyceride levels and 10% had low HDL-cholesterol levels. Among those with type 2 diabetes, 65% had elevated triglyceride levels, 60% had low HDL-cholesterol levels and 35% had elevated apolipoprotein levels [68]. Alarmingly, in the TODAY study, the 15 year cumulative incidence of dyslipidaemia and hypertension was 51.6% and 67.5%, respectively [70].

Of note, a sexual dimorphism exists for diabetes-associated cardiometabolic risk factors and complications, which tend to be more common in girls than in boys [63, 69]. During adolescence, girls experience more severe insulin resistance, higher HbA_1c_ levels and BMI and a greater prevalence of dyslipidaemia than boys [71, 72], which can contribute to their higher risk of complications [66, 73]. Adolescent girls with albuminuria also show higher testosterone levels, a higher free androgen index and reduced sex hormone-binding globulin levels than those without this complication, suggesting that increased androgen production may contribute to the early signs of vascular complications in girls [73]. Lower levels of IGF-1 in girls with type 1 diabetes have also been associated with the development of albuminuria [73].

The prevalence of vascular complications is higher in youth with type 1 diabetes from LMICs than those from HICs. In a cross-sectional study from Tanzania, 41.9% of youth aged 5–19 years had at least one microvascular complication. Of those presenting with complications, 26% had two complications and 4.6% had three [74]. In another Tanzanian cohort, early signs of diabetic retinopathy and kidney disease were seen even in children aged 5–10 years after a short duration of diabetes [75]. Similar data have been reported from Sudan, where a cross-sectional study found that 58% of youth aged 10–18 years had diabetic kidney disease, retinopathy or both after a median diabetes duration of 7 years [76]. A cohort study in Ethiopia found that 25% of children had at least one microvascular complication within 5 years of type 1 diabetes diagnosis, and 75% had at least one complication by 8 years [77]. Finally, according to 2014 data from India, regardless of the type of diabetes, half of the study population aged 10–25 years developed diabetic retinopathy within 10–12 years of diabetes diagnosis [78].

Ethnic disparities play a significant role in influencing the risk of complications, with non-Hispanic Black individuals with type 1 diabetes having a 4.5 fold increased risk of above-target HbA_1c_ and a twofold increase in systolic blood pressure compared with their non-Hispanic White counterparts [79]. These ethnic differences persist even after adjusting for age and socioeconomic status, suggesting that other underlying mechanisms, such as genetic predisposition, cultural lifestyle differences, healthcare access, diet, physical activity levels and psychosocial stressors, might play a role [80].

Social determinants of health affect diabetes and its related outcomes [81, 82]. Factors such as low socioeconomic status, food insecurity, unstable housing, transport challenges, limited access to healthcare and social isolation have all been found to negatively impact overall health and diabetes-related outcomes [82]. Higher rates of hyperglycaemia, albuminuria, dyslipidaemia and hypertension have been associated with deprivation in youth, irrespective of diabetes type [83, 84].

### Risk of mortality in youth with diabetes

An earlier age at diagnosis of type 2 diabetes is associated with higher all-cause mortality [63]. A meta-analysis of around 1.3 million youth diagnosed with type 2 diabetes reported a 4% decreased risk of all-cause mortality for each 1 year increase in age at diagnosis after controlling for current age [61]. In addition, the SEARCH study reported 1.5 times higher standardised mortality ratios for youth-onset type 2 diabetes compared with type 1 diabetes (2.3 vs 1.5) [85]. The same study also highlighted that excess mortality was highest among racial and ethnic minority groups and those younger than 25 years at the time of death. Of note, only 9.1% of deaths in youth with type 2 diabetes had diabetes-related factors as the underlying cause, with external factors (injury, assault, motor vehicle accident) being the most common cause of death.

According to GBD 2019 data, type 1 diabetes-related mortality rates increased between 1990 and 1999, after which a steady decline was noticed. Countries with the highest SDI have the lowest mortality rates (0.05 per 100,000), whereas those with the lowest SDI show the highest mortality rates (0.5 per 100,000) [42, 86]. Globally, there is a huge gap in remaining life expectancy (40 vs 64 years) between newly diagnosed 10-year-old children with type 1 diabetes and their peers without type 1 diabetes. This gap ranges from 46 years in low-income countries (LICs) to 36 years in LMICs and 11 years in HICs [45]. The marked reduction in life expectancy in LICs and LMICs is related to higher mortality rates resulting from acute and chronic complications such as diabetic ketoacidosis, hypoglycaemia, infections and end-stage renal failure. These outcomes reflect differences in healthcare provision, health infrastructure and accessibility to affordable diabetes supplies such as insulin, glucose monitoring systems and diabetes education between LICs/LMICs and HICs. When the governments of LICs and LMICs are unable to provide comprehensive care, studies have shown that scaling up ‘minimal’ diabetes care to even ‘intermediate’-level care (basal–bolus regimen, two to three blood glucose tests per day, regular HbA_1c_ testing, complications screening, age-appropriate diabetes education, peer and school support and 24 h emergency call support) can lead to significant reductions in complications and mortality rates and is very cost-effective [87, 88].

## How to decrease the burden of early-onset metabolic diseases

**Fig. 4:**
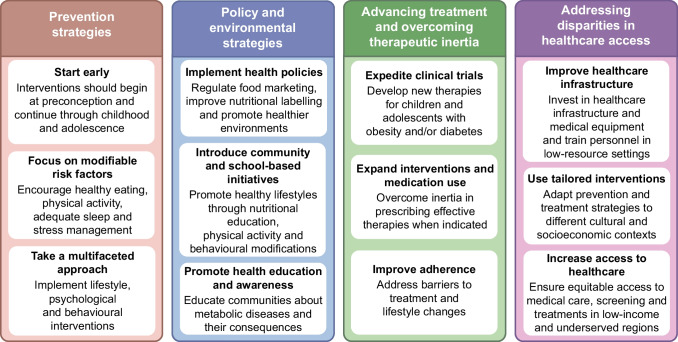
Strategies to reduce the burden of youth-onset metabolic diseases. This figure is available as part of a downloadable slideset

The increasing incidence and severity of youth-onset obesity and type 1 and type 2 diabetes raises concerns about the growing number of individuals at risk of developing cardiometabolic complications throughout their lifespan and experiencing disability and reduced life expectancy [3]. Addressing this issue requires a multifaceted approach that includes early interventions, improved healthcare policies, enhanced treatment strategies and addressing disparities in healthcare access (Fig. 4).

To control the spread of metabolic diseases in youth worldwide, interventions should begin early, starting from pre-conception and continuing through infancy, childhood and adolescence [89]. Preventive measures should focus on modifiable risk factors, including diet, physical activity levels, sleep and stress management [90]. A multidisciplinary approach involving lifestyle and behavioural interventions is essential to instil healthy habits early, supporting long-term health and reducing the risk of chronic diseases later in life [91].

Health policies are essential for preventing early-onset metabolic disorders, fostering a healthier environment through measures such as regulating food marketing, enhancing nutritional labelling and promoting healthy eating. A notable example is the increased taxation on sugar-sweetened beverages, which has proven effective in reducing obesity rates [92]. Policies should also encourage active school environments and support community-based initiatives that aim to make physical activity accessible and enjoyable for all children [93]. Raising professional and public awareness of metabolic diseases and their health implications is another critical step in preventing the escalation of this problem [94].

Managing youth-onset metabolic disorders and mitigating the associated complications also requires the development of more effective treatments as well as strategies to overcome barriers to adherence, which is suboptimal among youth [95]. Therapeutic inertia—the reluctance to initiate or escalate necessary treatments—should also be addressed. Many healthcare professionals hesitate to prescribe medications such as metformin, glucagon-like peptide-1 (GLP-1) receptor agonists, antihypertensives and lipid-lowering drugs due to concerns over limited long-term safety data, lack of clear guidelines, inadequate training and resource constraints [96]. Expediting clinical trials of new interventions in youth with obesity and/or diabetes is crucial, particularly given the success of therapies such as sodium–glucose cotransporter 2 inhibitors and GLP-1 receptor agonists in adults. Expanding treatment options for children and adolescents is critical, as current strategies are often inadequate.

Further understanding of ethnic and socioeconomic disparities is also crucial to develop targeted interventions that address the unique needs of diverse populations. Because of the differences in attributable risk factors for early-onset metabolic diseases across regions and countries [33], specific policies should be established to manage this epidemic more effectively.

Addressing inequalities in access to healthcare is vital, as the burden of metabolic diseases remains disproportionately high in LMICs [97]. This disparity is partly caused by delayed diagnosis of metabolic diseases and related complications, due to limited and inadequate infrastructure and diagnostic capabilities [97]. In addition, treatment strategies for obesity and diabetes available in HICs are often unaffordable in LMICs. Alarmingly, access to insulin remains limited in some LMICs, and diabetes technologies are largely inaccessible in low-resource settings due to their cost [97]. Therefore, clear strategies and increased investment are needed to improve diagnostics and access to insulin and other essential treatments for young people living in LMICs.

## Conclusions

The increasing rates of metabolic diseases among young people, along with regional, ethnic and socioeconomic disparities, raise significant concerns about their short- and long-term health impacts. Early targeted interventions are crucial to prevent the onset and progression of these diseases.

Tackling youth-onset metabolic diseases requires a global, multidisciplinary and policy-driven approach. From early prevention to equitable healthcare access, comprehensive strategies must be implemented to reduce the burden of these conditions and improve long-term health outcomes. Additionally, tailored preventive and treatment strategies that consider cultural contexts, socioeconomic backgrounds and available resources are essential to effectively manage these diseases across diverse settings.

## Supplementary Information

Below is the link to the electronic supplementary material.

Slideset of figures (PPTX 1.10 MB)

## Abbreviations

DALY: Disability-adjusted life-year
GBD: Global Burden of Disease Study
GLP-1: Glucagon-like peptide-1
HICs: High-income countries
LICs: Low-income countries
LMICs: Low- and middle-income countries
SDI: Sociodemographic index
TODAY: Treatment Options for type 2 Diabetes in Adolescents and Youth
