# Supplementary material for: Metabolic disorders in young people around the world
Source: Diabetologia. 2025 Jun 17;68(11):2374–85. doi: 10.1007/s00125-025-06450-2 (PMC12534309; doi:10.1007/s00125-025-06450-2)
Supplement: Supplementary file 1 — Slideset of figures (PPTX 1.10 MB) [file 125_2025_6450_MOESM1_ESM.pptx]

## Slide 1
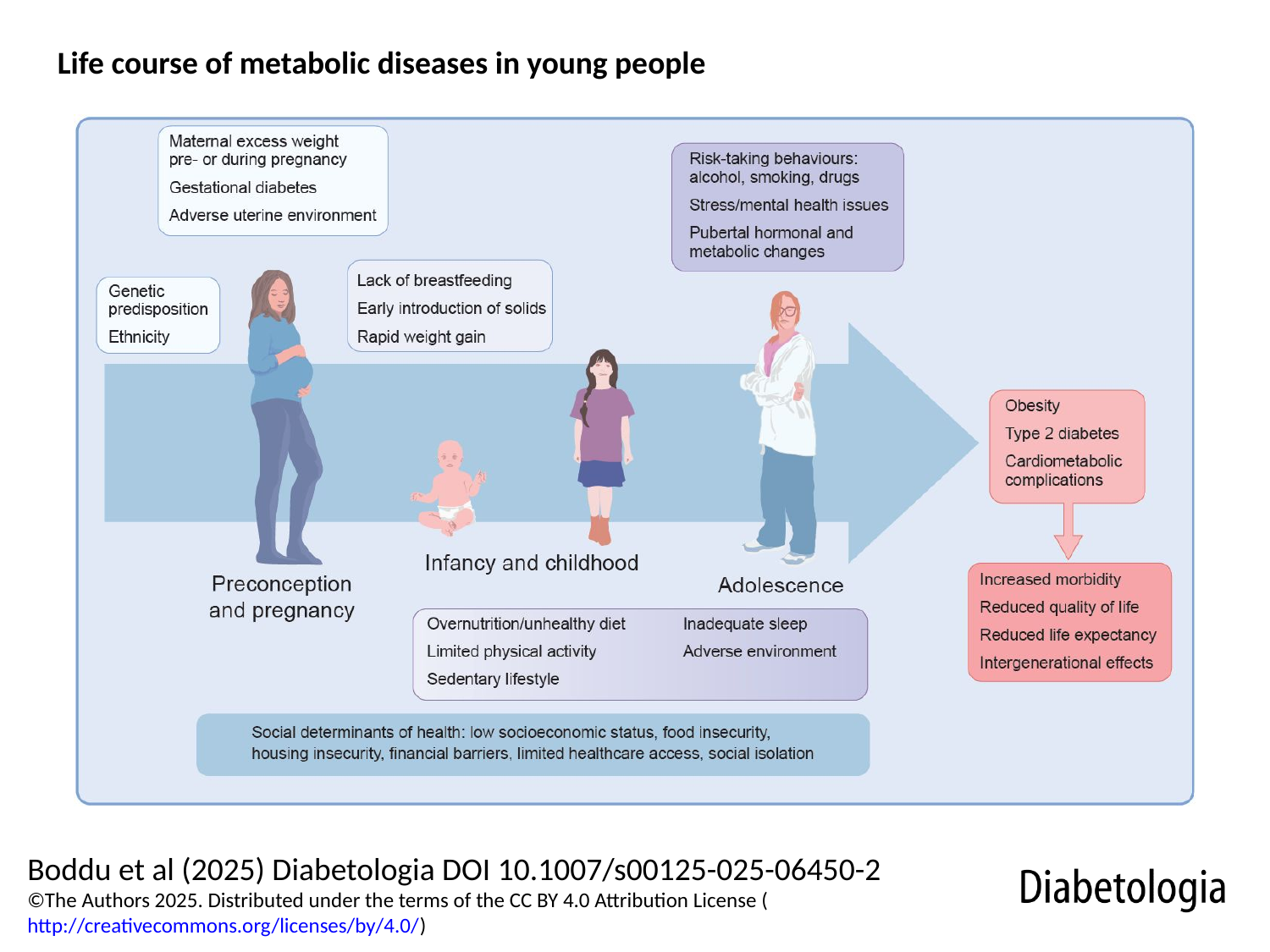

Life course of metabolic diseases in young people
Boddu et al (2025) Diabetologia DOI 10.1007/s00125-025-06450-2
©The Authors 2025. Distributed under the terms of the CC BY 4.0 Attribution License (http://creativecommons.org/licenses/by/4.0/)

## Slide 2
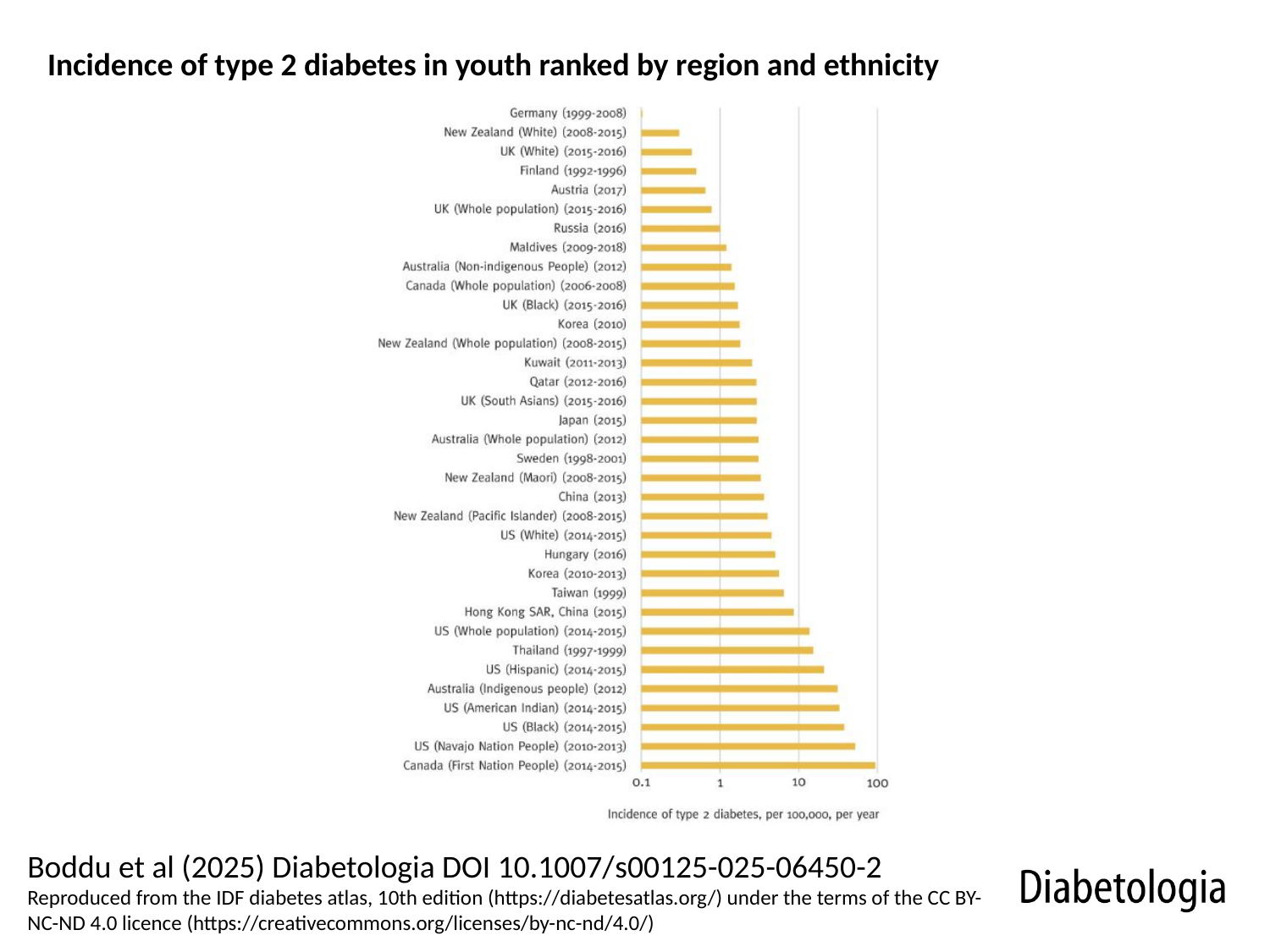

Incidence of type 2 diabetes in youth ranked by region and ethnicity
Boddu et al (2025) Diabetologia DOI 10.1007/s00125-025-06450-2
Reproduced from the IDF diabetes atlas, 10th edition (https://diabetesatlas.org/) under the terms of the CC BY-NC-ND 4.0 licence (https://creativecommons.org/licenses/by-nc-nd/4.0/)

## Slide 3
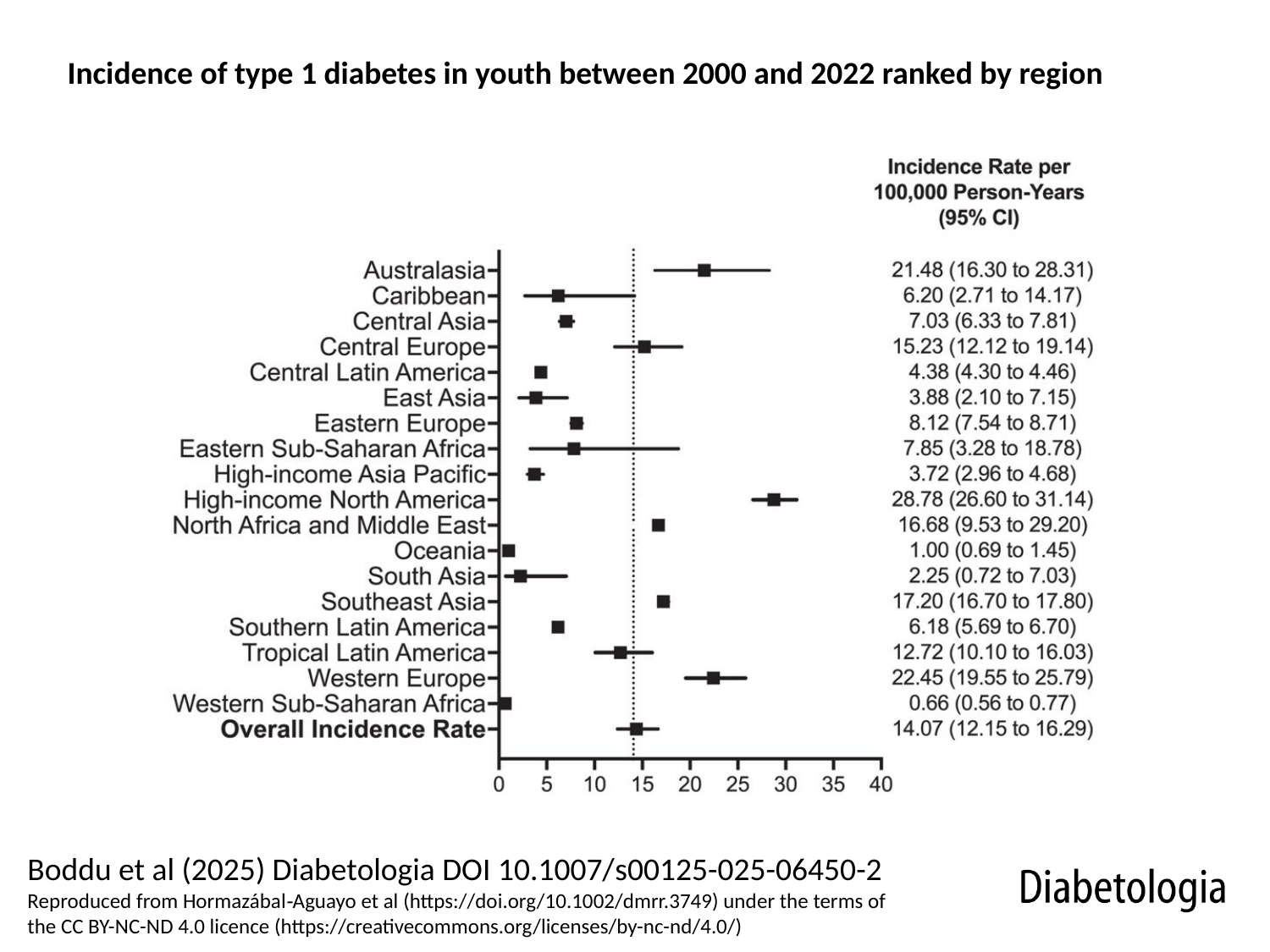

Incidence of type 1 diabetes in youth between 2000 and 2022 ranked by region
Boddu et al (2025) Diabetologia DOI 10.1007/s00125-025-06450-2
Reproduced from Hormazábal‐Aguayo et al (https://doi.org/10.1002/dmrr.3749) under the terms of the CC BY-NC-ND 4.0 licence (https://creativecommons.org/licenses/by-nc-nd/4.0/)

## Slide 4
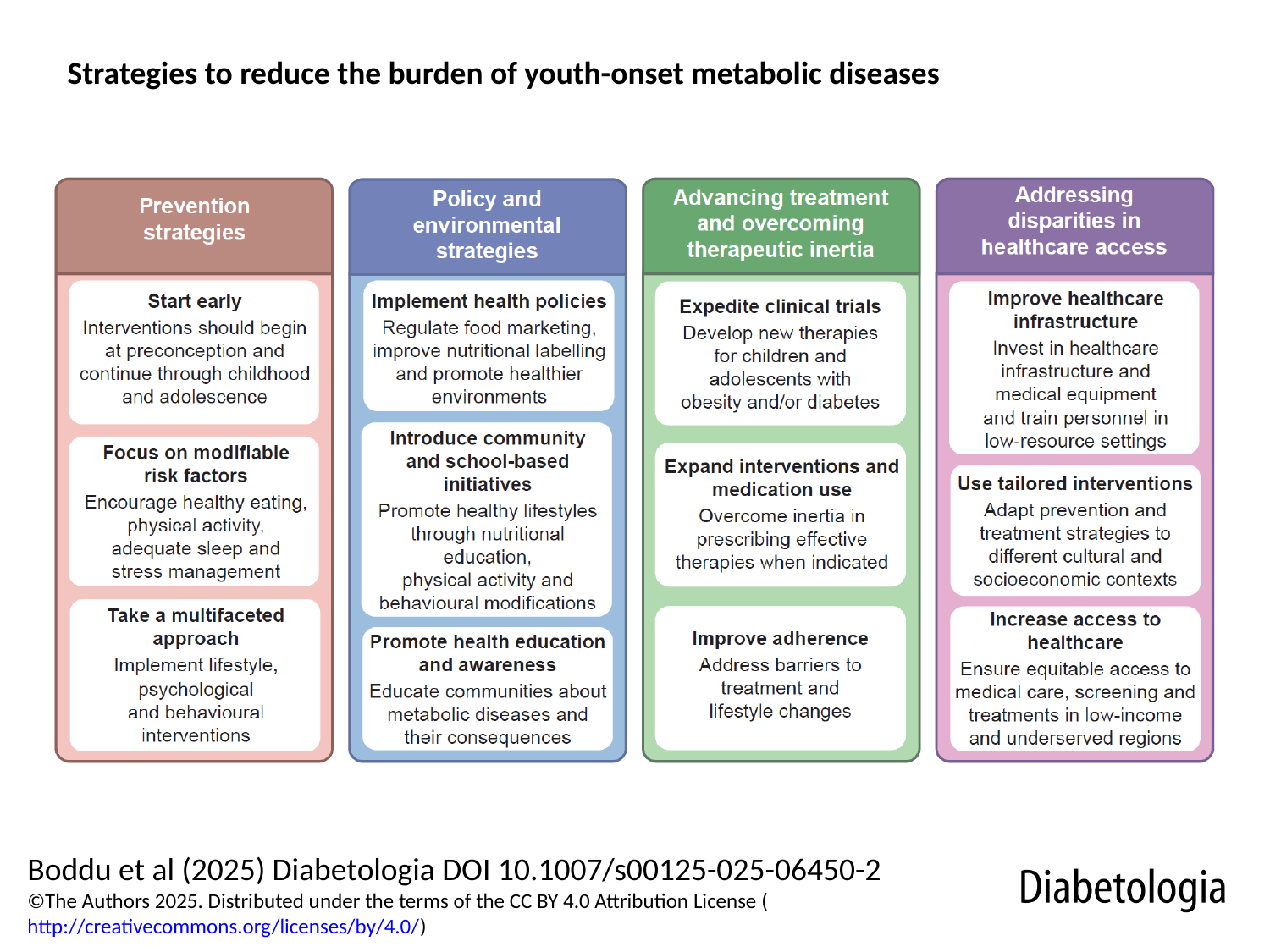

Strategies to reduce the burden of youth-onset metabolic diseases
Boddu et al (2025) Diabetologia DOI 10.1007/s00125-025-06450-2
©The Authors 2025. Distributed under the terms of the CC BY 4.0 Attribution License (http://creativecommons.org/licenses/by/4.0/)
